# Attitudes towards chiropractic: an analysis of written comments from a survey of north american orthopaedic surgeons

**DOI:** 10.1186/2045-709X-19-25

**Published:** 2011-10-04

**Authors:** Jason W Busse, Janey Jim, Craig Jacobs, Trung Ngo, Robert Rodine, David Torrance, Abhaya V Kulkarni, Brad Petrisor, Brian Drew, Mohit Bhandari

**Affiliations:** 1 The Institute for Work & Health, 481 University Avenue, Suite 800, Toronto, Ontario M5G 2E9, Canada; 2 The Department of Clinical Epidemiology and Biostatistics, McMaster University, 1280 Main Street West, Hamilton, ON L8S 4K1, Canada; 3 The Canadian Memorial Chiropractic College, 6100 Leslie Street Toronto, ON M2H 3J1, Canada; 4 The Division of Population Health Sciences, Hospital for Sick Children, Room 1503, 555 University Ave, Toronto, ON M5G 1X8, Canada; 5 The Department of Surgery, McMaster University, 1280 Main Street West, Hamilton, Ontario L8S 4L8, Canada

**Keywords:** orthopaedics, chiropractic, attitude of health personnel, survey

## Abstract

**Background:**

There is increasing interest by chiropractors in North America regarding integration into mainstream healthcare; however, there is limited information about attitudes towards the profession among conventional healthcare providers, including orthopaedic surgeons.

**Methods:**

We administered a 43-item cross-sectional survey to 1000 Canadian and American orthopaedic surgeons that inquired about demographic variables and their attitudes towards chiropractic. Our survey included an option for respondants to include written comments, and our present analysis is restricted to these comments. Two reviewers, independantly and in duplicate, coded all written comments using thematic analysis.

**Results:**

487 surgeons completed the survey (response rate 49%), and 174 provided written comments. Our analysis revealed 8 themes and 24 sub-themes represented in surgeons' comments. Reported themes were: variability amongst chiropractors (n = 55); concerns with chiropractic treatment (n = 54); areas where chiropractic is perceived as effective (n = 43); unethical behavior (n = 43); patient interaction (n = 36); the scientific basis of chiropractic (n = 26); personal experiences with chiropractic (n = 21); and chiropractic training (n = 18). Common sub-themes endorsed by surgeon's were diversity within the chiropractic profession as a barrier to increased interprofessional collaboration, endorsement for chiropractic treatment of musculoskeletal complaints, criticism for treatment of non-musculoskeletal complaints, and concern over whether chiropractic care was evidence-based.

**Conclusions:**

Our analysis identified a number of issues that will have to be considered by the chiropractic profession as part of its efforts to further integrate chiropractic into mainstream healthcare.

## Background

In 2006 the Chiropractic Strategic Planning Conference proposed a set of recommendations for advancing the chiropractic profession in North America [1]. These included interdisciplinary clinical training, the integration of chiropractic into mainstream healthcare, and increased interprofessional collaboration. Advancing these initiatives will require endorsement from healthcare professionals that attend to patient populations who also seek chiropractic care, which includes physical therapists, family physicians, physiatrists, and orthopaedic surgeons. It is helpful to establish the attitudes of these professional groups towards chiropractic in order to understand current levels of interaction and what barriers exist to increased interprofessional collaboration.

We recently surveyed 1000 North American orthopaedic surgeons (49% response rate) to inquire about their use of, and attitudes towards, chiropractic [2]. Approximately half of those surveyed (52%; 252 of 487) referred at least some patients for chiropractic care each year, and attitudes towards chiropractic ranged from very positive to extremely negative. In our adjusted generalized linear model, factors associated with more negative attitudes included older age and endorsement of the research literature, the media or medical school as a source of information regarding chiropractic. More positive attitudes were associated with endorsing a relationship with a specific chiropractor as a source of information regarding chiropractic. Our survey included an option for respondents to provide written comments; however, our initial publication did not allow for sufficient space to discuss this material in detail due to the amount of data provided. We therefore reviewed and synthesized this material in order to supplement our previously reported findings and to further inform current barriers to the integration of chiropractic into mainstream healthcare.

## Methods

We have reported details of our questionnaire development and administration elsewhere [2]. In brief, we developed a 43-item, English language questionnaire to examine orthopaedic surgeons' attitudes towards chiropractic. Our survey included 20-items that asked respondents to indicate their attitudes towards chiropractic - the chiropractic attitude questionnaire (CAQ). Each of the 20 questions comprising the CAQ is graded on a 5-point Likert scale, from 0 to 4. The responses are then summed to arrive at a total score ranging from 0 (most negative attitude towards chiropractic) to 80 (most positive attitude towards chiropractic). Our survey also included an option for surgeons to provide written comments regarding additional thoughts they may have regarding chiropractic.

From July 2006 to June 2007 we administered our survey to a random sample of 500 Canadian orthopaedic surgeons, and from July 2007 to June 2008 we administered the same survey to a random sample of 500 American orthopaedic surgeons. We administered surveys by fax, and all surgeons' offices were telephoned prior to sending a survey to confirm their presence and fax number. Participants were provided with a disclosure letter detailing the intent of the survey and explicit instructions that, should they choose not to complete the survey, they could check a box on the cover page indicating that they did not wish to participate and fax it back to our attention. At 4 and 8 weeks following the initial mailing, we re-faxed the questionnaire to all non-responders. We telephoned each office that received a 3^rd ^(final) survey prior to faxing in an effort to encourage completion of the instrument. The McMaster University Research Ethics Board approved our study.

### Statistical analysis

We have reported our analysis of respondent's survey data elsewhere [2] and the current analysis is restricted to a thematic analysis [3-5] of written comments provided by surgeons. In order to systematically review comments two reviewers (JWB and JJ) developed a coding system to categorize themes and sub-themes. We developed coding rules through discussion and after four major rounds of coding written comments, clusters around themes emerged that we used to build a coding tree. Each survey that provided written comments could contribute more than 1 theme or sub-theme, but each theme or sub-theme was only coded once in a single survey to address the issue of clustering. When the tree structure became stable, as evidenced by new articles generating no new codes, and disagreement among reviewers became minimal, we applied our coding strategy, independently and in duplicate, to all written comments. Disagreements were resolved through discussion to achieve consensus. We decided, *a priori*, only to present sub-themes that were endorsed by a minimum of 3 survey respondents. Our selection of quotations for presentation was guided by consensus among reviewers that selected statements were particularly informative, representative and succinct.

We generated frequencies for demographic characteristics and calculated mean CAQ scores for respondents who provided written comments and checked for differences between Canadian and American surgeons with an independent samples t-test and χ^2 ^test. We explored for differences in mean CAQ scores between survey respondents who provided written comments and those who did not to explore for the presence of an attitudinal bias. All comparisons were 2-tailed and we set our level of significance at p ≤ 0.01 to account for multiple comparisons. We performed all analyses using PASW Statistics 18.0 (IBM, New York, NY).

## Results & Discussion

Surgeons completed 487 of 1000 surveys, of which 174 (36% of respondents) provided written comments. Characteristics of surgeons that provided written comments are provided in Table 1. We did find evidence of attitudinal bias among surgeons who provided written comments; specifically, they were more likely to hold less positive attitudes towards chiropractic (mean difference in CAQ scores = -3.47; 95% confidence interval = -5.68 to -1.27; p = 0.002). We coded a total of 309 sub-themes from all written comments with an overall agreement of 80%. Our coding revealed 8 distinct themes and 24 sub-themes represented in surgeons' written comments (Table 2). A description of these themes and sub-themes, with representative quotes, follows.

**Table 1:** Demographic characteristics of respondents who provided written comments

	Currently Practicing in Canada	Currently Practicing in the United States
N	80	94

Age, mean (SD) *	51.3 (9.7)	55.0 (9.6)

Gender, n (%)		
Male	76 (95%)	90 (96%)
Female	4 (5%)	4 (4%)

Years in practice, n (%)		
<5 years *	8 (10%)	0
5 to 10 years *	14 (18%)	5 (5%)
11 to 20 years	23 (29%)	35 (37%)
>20 years	35 (44%)	54 (57%)

Practice environment, n (%)^†^		
Community	32 (40%)	27 (29%)
Hospital-based *	24 (30%)	4 (4%)
Multidisciplinary	8 (10%)	2 (2%)
Private practice *	30 (38%)	70 (75%)
Academic *	38 (48%)	7 (8%)

Patient population, n (%)		
Adult *	45 (56%)	25 (27%)
Pediatric	8 (10%)	7 (7%)
Adult & Pediatric *	27 (34%)	62 (66%)

Clinical area, n (%)^†^		
Spine	24 (30%)	26 (28%)
Upper extremity	25 (31%)	45 (48%)
Reconstructive/Arthroplasty	45 (56%)	43 (46%)
Foot & Ankle	22 (28%)	27 (29%)
Oncology	4 (5%)	2 (2%)
Sports injuries *	30 (38%)	53 (57%)
Trauma	33 (41%)	41 (44%)
Other	11 (14%)	15 (16%)

CAQ score, mean (SD)	31.3 (13.0)	33.4 (12.4)

* = differences between groups are statistically significant (p < 0.01)

^† ^= total percentage is >100% as respondents could choose more than one option

**Table 2:** Coding tree for written comments recorded in surveys

Themes		Sub-themes (n)	Number of Endorsements
	n		

Variability amongst chiropractors	0	No distinct sub-themes were identified	55

Areas where chiropractic is perceived as effective	4	Mechanical low back pain	16
		Musculoskeletal disorders	16
		Non-surgical injuries	8
		Spinal complaints	3

Concerns with chiropractic treatment	6	Chiropractic care is of marginal or no benefit	13
		Non-musculoskeletal complaints	12
		Serious adverse events	11
		General medical care	8
		Non-spinal complaints	7
		Structural scoliosis	3

Patient Interaction	4	Chiropractic terminology is misleading	17
		Chiropractors make false or exaggerated claims	13
		Chiropractors spend considerable time with patients	6

Chiropractic training	2	Chiropractors are poorly trained	10
		Other therapists can provide spinal manipulation	8

Unethical Behavior	4	Chiropractors treat excessively	18
		Chiropractors are overly financially motivated	15
		General ethical concerns	5
		Chiropractic requires stronger regulatory oversight	5

Scientific basis of chiropractic	2	Chiropractic is unscientific	20
		More chiropractic research is needed	6
			

Personal experiences with chiropractic	3	Positive experiences	13
		Negative experiences	4
		Uncertainty as to the role of chiropractic	4

### Variability amongst chiropractors

The most commonly endorsed theme was diversity within the chiropractic profession: "*Wide range of practice - some are evidence based...others do all sorts of crazy stuff*". Eleven respondents acknowledged difficulty in answering our survey, in that their responses to a number of questions would depend on the type of chiropractor under consideration: "*Obviously not all [chiropractors] are the same...This questionnaire relates to the majority of [chiropractors], not all*". Fourteen respondents dichotomized by 'good' and 'bad' chiropractors, and 10 acknowledged similar limitations in orthopaedics: "*There are good chiropractors and bad orthopaedic [surgeons] and vice versa*", and: "*I explain to my patients that there are excellent chiropractors just like there are bad orthopaedic surgeons. But I wouldn't pick one out of a book or from an ad*". Six respondents felt that diversity among practitioners was harmful to the chiropractic profession: "*I think that chiropractic is hurt by the fringe treatments that some offer*", and: "*... some unethical chiropractors have given the profession a bad name*".

### Areas where chiropractic treatment is perceived as effective

Three respondents felt that chiropractic care for spinal complaints was effective, and 16 endorsed care specifically for low back pain: "*I feel chiropractic can be beneficial in helping mechanical low back pain*", and: "*I find their usefulness is limited to lumbar mechanical back pain care*". Sixteen respondents endorsed an expanded role for the treatment of musculoskeletal disorders in general: "*I think chiropractic care is beneficial for musculoskeletal pain if there are no risk factors*", and: "*chiropractors have a role in chronic musculoskeletal pain control*". Eight surgeons suggested a broad role in the management of non-surgical injuries: "*I have a very good relationship with a [chiropractor]...He has helped many members of our sports teams with hands on care of acute and chronic non surgical injuries*".

### Areas where chiropractic treatment is perceived as unhelpful or problematic

Fifty-four surgeons noted concerns with different aspects of chiropractic care. Some focused on non-spinal conditions, and 3 with chiropractic management of structural scoliosis; however, most respondents (n = 13) in this category felt that chiropractic care provided short-term benefit only or was ineffective: "*No objective long term benefits*", "*Chiropractors understand that a majority of back or neck strain ailments will resolve with or without 'therapy'*", and: "*It is only after all the therapy visits that do not require pre-authorization or the insurer notes no improvement that the worker gets referred for an orthopedic evaluation*".

The second most commonly endorsed concern (n = 12) took issue with treatment of non-musculoskeletal complaints: "*I have a huge problem with chiropractors claiming to treat asthma and ear infections among other non-musculoskeletal problems*", and: "*most chiropractors (in my view) are very helpful for spinal complaints. Manipulation for asthma, high blood pressure, breech presentation is quackery - not evidence based. We all need to stick to what we do best*".

Eleven respondents raised concerns regarding the provision of general medical care by chiropractors: "*Chiropractors are dangerous when they portray themselves as replacements for patients' primary care physicians*", and: "*There are 2 breeds of chiropractors, one who treats only musculoskeletal problems and knows their limitations and does not try to treat everything with manipulation. Then there is the other group who feel they are family physicians which they are not and try to treat all problems like asthma or infections with manipulation*". Eleven respondents expressed concerns with adverse events associated with chiropractic care: "*In the last 2 years I have seen 2 patients with vertebral artery dissection within 10 days of chiropractic neck manipulation*", "*Have on several occasions done emergency surgery resulting from chiropractic management*", and: "*In the orthopedic department in which I worked we saw paraplegics produced by spinal manipulation by chiropractors*".

### Patient Interaction

Surgeons were largely critical of chiropractors' interactions with patients. Most concerns were directed towards chiropractic terminology, which respondents felt was often misleading or incorrect: "*The concept of 'adjusting subluxations' is nonsensical. 'Adjusting' hips and sacroiliac joints is nonsensical*", "*Why do chiropractors cling to an antiquated theory as inappropriate as 'the humors'?*", "*Every patient is told that one leg is longer than the other as the root of their medical problems*", and: "*When chiropractors change the principal that 'all disease emanates from the spine and can be cured by manipulation' they will be more welcome in the medical community and hospitals. It is no question that they are the best at manipulation*".

A number of respondents felt that chiropractors often made false or exaggerated claims: "*False information given to patients - I had a cerebral palsy child going to a chiropractor for 5 years to improve his gait!*", and: "*...much of their 'explanations' are based on grains of truth that are distorted to support their claims*"; however, 6 did acknowledge non-specific benefits of the clinical encounter: "*Chiropractors are very good at taking care of the 'worried well' patients*", and: "*Very complimentary (sic) to musculoskeletal care - they often talk to patients for great lengths of time while treating, unlike ourselves*".

### Chiropractic training

Ten surgeons noted concerns with chiropractic training, with two respondents noting a lack of standardization and 2 mentioning specific schools: "*Life chiropractors are scary*", and "*I visited the Canadian Memorial Chiropractic College in Toronto. I was impressed with the similarity of their basic science curriculum - same books as used in medicine - and the extent of information covering all symptoms. This does not seem to be the case for all schools in North America*". Two respondents noted their perception that "*younger [chiropractors] are better prepared and trained than their older colleagues*", while 2 others reported concern regarding "*a lack of diagnostic skills*". Two other surgeons noted their experiences with both well and poorly trained chiropractors: "*I personally use and refer to several extremely well trained [chiropractors] in my area; however, there are much more poorly trained [chiropractors] than well trained [chiropractors]. Overall, appropriate treatment by well trained [chiropractors] is very valuable*".

Eight surgeons noted their perception that the helpful aspects of chiropractic care could largely be provided by other therapists: "*All chiropractic care could be better managed by physiotherapy and occupational therapy*", "*Manipulative therapy is not the exclusive domain of chiropractors. Many physical therapists and athletic therapists are also skilled with that technique and deserve equal recognition*", and: "*Manipulation of back for mechanical back pain can be done by physiotherapists. There is no need for chiropractors*".

### Unethical Behavior

Concerns over excessive treatment by chiropractors were raised by 18 respondents: "*Major problem as I see it, chiropractors tend to place patients on an 'x amount of treatment' plan such as 20 visits. In my opinion they need to perform the service and see if the patient responds and proceed from there. Not just say 'you need an x amount of treatment' plan*", "*The concept of repetitive on-going care (maintenance/prevention) is completely contrary to common sense and science. This is what I object to*", and: "*Many chiropractors take advantage with the lawyers on auto accident cases. Over treating until the medical payments are exhausted*". Fifteen surgeons also noted concerns that some chiropractors may be overly financially motivated: "*I believe that most chiropractors are ethical...The trouble is that there are many who team up with unethical legal/rehab facilities and abuse the system for personal gain, partly at the detriment of patient care*", and: "*Money is the only driving force behind many chiropractor's treatment plans*".

Five surgeons noted more general concerns with unethical behavior: "*Too many chiropractors engage in unethical practices*", and "*When I started practicing 28 years ago several chiropractors in the area would refer me patients and I'd refer them back. That pattern ended 15 years ago for unknown reasons except many chiropractors in this area are now employees of entities that have sales quotas, profit goals, and widening 'healthcare' ambitions that seemingly come first to patient care*". Five respondents advised that the chiropractic profession requires stronger oversight: "*Their profession needs more careful government scrutinizing and control*", "*I think that most chiropractors are honest...unfortunately they are overshadowed by dishonest ones...my opinion is that chiropractors should establish an honest ethics committee that [medical doctors can report] despicable behavior*", and: "*More self policing by chiropractors at the state level would help them immensely*".

### Scientific basis of chiropractic

Twenty respondents took issue with the scientific basis underlying chiropractic: "*Most students now have undergraduate degrees and are relatively well educated in basic sciences in their first 2 years (preclinical). The problem begins when they move into their clinical years where education becomes 'indoctrination'. Extracts from basic science are selectively constructed into a theory of practice with little if any real scientific basis*", "*Chiropractic care would be better accepted in medical circles if it were evidence based/scientific and realistic in its' claims and applications to patients*", and: "*There is no place for chiropractors to work side by side with scientific medical practitioners*". Six surgeons advocated for more research into chiropractic treatment: "*We need some evidence based chiropractic. Publications in orthopedic journals or medical journals with peer review*", and: "*[Chiropractors] need to produce more clinical studies of effectiveness*".

### Personal experiences with chiropractic

Thirteen respondents reported positive personal experiences with chiropractic: "*Chiropractic has been a valuable accessory to my practice. In particular dealing with international, national and professional athletes*", "*I have a very good relationship with a [chiropractor]...given a diagnosis is established, his care is very efficacious!*", and: "*I go to one myself for neck and back problems*". However, four surgeons noted negative experiences: "*I have personally cared for at least 2 patients that had wrist tendonitis that were off work for *≥*1 year [attending chiropractic care] that only needed 1 shot to be cured*", and another 4 expressed uncertainty as to the role of chiropractic in healthcare: "*I need more knowledge to utilize chiropractors better!*", and: "*Unclear at this point what their role is*".

#### Summary of Findings

We found that written comments by orthopaedic surgeons most frequently endorsed diversity within the chiropractic profession as a barrier to increased interprofessional collaboration. Surgeons identified that training, diagnostic ability and treatment skills were highly variable within the chiropractic profession, and highlighted that unethical behaviors by some affected perceptions of the profession as a whole. Respondents largely felt that chiropractors provided helpful care for musculoskeletal complaints, and particularly mechanical low back pain, but were concerned about the potential for adverse events and were very critical of chiropractic management of non-musculoskeletal conditions. Respondents called for greater integration of evidence-based practices within chiropractic, more chiropractic-related research, and increased oversight by regulatory bodies.

Strengths of our study include a detailed assessment of all written comments, conducted independently and in duplicate and guided by a comprehensive coding strategy; however, our study does have limitations. Surgeons who provided written comments tended to have more negative attitudes towards chiropractic. Furthermore, we coded all unique themes or sub-themes from each survey which means that some respondents contributed more content to our analysis than others. Generalizability of our findings should be explored in a representative sample of North American orthopaedic surgeons.

Respondents' general perceptions that chiropractic treatment, primarily defined by joint manipulation, has been found to be effective for some musculoskeletal complaints (particularly mechanical low back pain), but not for non-musculoskeletal conditions, is generally consistent with current evidence [6-14]. Despite some respondent's concerns, current evidence also suggests that chiropractic care is not causally related to serious adverse events, specifically vertebrobasilar artery stroke [15,16]; however, efforts are currently underway to better establish the risks associated with spinal manipulation [17]. Diversity within the chiropractic profession has been previously reported [18,19], and some practitioners have concluded that "for every chiropractor there is an equal and opposite chiropractor" [20].

Unethical practices by some chiropractors have also been acknowledged by the profession. In 2008 the Ontario Chiropractic Association conducted a membership survey (n = 2775, 34% response rate) which found the majority (67%) "felt that the practices of their peers are in some way negatively influencing perceptions of the profession" [21]. Opinion leaders within the chiropractic profession have noted the need for more research and greater incorporation of evidence-based practices [22-24]; however, internal research capacity is limited. A 2008 survey of all members of the Canadian Chiropractic Association (~6000 members; 684 responded) found that considerably less than 1% of the profession was engaged in conducting research [25].

Our findings suggest that there are a number of concerns held by North American orthopaedic surgeons that should be addressed as part of any efforts to facilitate greater integration of chiropractic into mainstream healthcare. Our analysis of written comments provided by surgeons emphasized the importance of a number of items that we had incorporated into our survey. These include chiropractic's role in managing musculoskeletal and non-musculoskeletal complaints, perceptions of excessive treatment by chiropractors, and whether or not chiropractic is perceived as evidence-based. New items suggested by our findings were diversity within the chiropractic profession as a barrier to interprofessional collaboration, perceptions of chiropractors as primary care providers, questions regarding where chiropractic care may be integrated into mainstream healthcare, and general concerns regarding adverse events associated with chiropractic care.

## Conclusions

Our analysis of written comments provided by North American orthopaedic surgeons who responded to our survey identified a number of issues that will have to be considered by the chiropractic profession as part of its efforts to further integrate chiropractic into mainstream healthcare. These include: diversity within the chiropractic profession as a barrier to interprofessional collaboration, chiropractic's role in managing musculoskeletal and non-musculoskeletal complaints, perceptions of excessive treatment by chiropractors, whether or not chiropractic is evidence-based, whether or not chiropractors are primary care providers, where chiropractic care may be integrated into mainstream healthcare, and general concerns regarding adverse events associated with chiropractic care.

## Competing interests

The authors declare that they have no competing interests.

## Authors' contributions

JWB conceived of and coordinated the study, performed the statistical analysis, and drafted the initial manuscript. CJ, TN, RR and DT administered the surveys and telephoned surgeons' offices. JWB and JJ coded all data. All authors contributed to survey design, and read and approved the final manuscript.
